# Prevalence and Risk Factors of COVID-19 Symptoms among U.S. Adults with Allergies

**DOI:** 10.3390/ijerph18052231

**Published:** 2021-02-24

**Authors:** Marlene Camacho-Rivera, Jessica Yasmine Islam, Denise Christina Vidot, Sunit Jariwala

**Affiliations:** 1 Department of Community Health Sciences, SUNY Downstate Health Sciences University, Brooklyn, NY 11203, USA; 2 UNC Lineberger Comprehensive Cancer Center, School of Medicine, UNC Chapel Hill, Chapel Hill, NC 27514, USA; islamjy@email.unc.edu; 3 Sylvester Comprehensive Cancer Center, School of Nursing and Health Studies, University of Miami, Miami, FL 33146, USA; dvidot@med.miami.edu; 4 Division of Allergy/Immunology, Albert Einstein College of Medicine and Montefiore Medical Center, Bronx, NY 10461, USA; SJARIWAL@montefiore.org

**Keywords:** allergy, COVID-19, coronavirus, SARS-CoV-2

## Abstract

Background: This study sought to evaluate COVID-19 associated physical and mental health symptoms among adults with allergies compared to the general U.S. adult population. Methods: Data for these analyses were obtained from the publicly available COVID-19 Household Impact Survey, which provides national and regional statistics about physical health, mental health, economic security, and social dynamics among U.S. adults (ages 18 and older). Data from 20–26 April 2020; 4–10 May 2020; and 30 May–8 June 2020 were included. Our primary outcomes for this analysis were physical and mental health symptoms experienced in the last seven days. The primary predictor was participants’ self-report of a physician diagnosis of an allergy. Results/Discussion: This study included 10,760 participants, of whom 44% self-reported having allergies. Adults with allergies were more likely to report physical symptoms compared to adults without allergies including fever (aOR 1.7, 95% CI 1.44–1.99), cough (aOR 1.9, 95% CI 1.60–2.26), shortness of breath (aOR 2.04, 95% CI 1.71–2.43), and loss of taste or sense of smell (aOR 1.9, 95% CI 1.58–2.28). Adults with allergies were more likely to report feeling nervous (cOR 1.34, 95% CI 1.13, 1.60), depressed (cOR 1.32, 95% CI 1.11–1.57), lonely (cOR 1.23, 95% CI 1.04–1.47), hopeless (cOR 1.44, 95% CI 1.21–1.72), or having physical reactions when thinking about COVID-19 pandemic (cOR 2.01, 95% CI 1.44–2.82), compared to those without allergies. During the COVID-19 pandemic, adults with allergies are more likely to report physical and mental health symptoms compared to individuals without allergies. These findings have important implications for diagnostic and treatment challenges for allergy physicians.

## 1. Introduction

According to international epidemiological and clinical studies, adults with existing chronic diseases are at highest risk of morbidity and mortality related to COVID-19 [1,2,3,4]. Several physical symptoms have been attributed as common presentations of COVID-19 including fever, cough, sore throat, and muscle or body aches [2]. In addition to the physical impact of the COVID-19 pandemic, the mental health of adults has been highly impacted [5,6,7,8,9,10,11,12]. Population-based data on the physical and mental health impacts of COVID-19 among United States (U.S.) adults with allergies are needed. Our objective was to evaluate COVID-19 associated physical and mental health symptoms experienced in the past seven days among adults with allergies compared to the general U.S. adult population. We hypothesized that adults with allergies would be more likely to experience physical and mental health related symptoms than those without allergies during the COVID-19 pandemic period. 

## 2. Methods

### 2.1. COVID-19 Impact Survey

Data for these analyses were obtained from the publicly available COVID-19 Household Impact Survey, conducted by the non-partisan and objective research organization NORC at the University of Chicago for the Data Foundation. The COVID-19 Household Impact Survey is a philanthropic effort to provide national and regional statistics about physical health, mental health, economic security, and social dynamics in the U.S. [13]. The survey is designed to provide weekly estimates of the U.S. adult (ages 18 and older) household population nationwide. Currently, data from Week 1 (20–26 April 2020), Week 2 (4–10 May 2020), and Week 3 (30 May–8 June 2020) are available, which were merged for this analysis. As the AmeriSpeak^®^ analytic sample of the COVID-19 Impact Survey was derived from de-identified publicly available data, Institutional Review Board approval was not required for this study.

### 2.2. AmeriSpeak Sample

Funded and operated by NORC at the University of Chicago, AmeriSpeak^®^ is a probability-based panel designed to be representative of the US household population. During the initial recruitment phase of the AmeriSpeak panel for the COVID Impact Survey, randomly selected US households were sampled using area probability and address-based sampling. These sampled households were then contacted by US mail, telephone, and field interviewers (face to face). The panel provides sample coverage of approximately 97% of the U.S. household population. Those excluded from the sample include people with P.O. Box only addresses, some addresses not listed in the USPS Delivery Sequence File, and some newly constructed dwellings. While most AmeriSpeak households participate in surveys via the Internet, households without web access were able to participate in AmeriSpeak surveys by telephone. Interviews were conducted in English and Spanish. Interviews were conducted with adults age 18 and over representing the 50 states and the District of Columbia. Panelists were offered a $5 monetary incentive for completing the survey. The number of participants invited and percentage of interviews completed by week are as follows: 11,133 invited with 19.7% interviews completed (Week 1); 8570 invited with 26.1% interviews completed (Week 2); and 10,373 invited with 19.7% interviews completed (Week 3). The analytic sample includes 10,760 adults nationwide and is weighted to reflect the US population of adults aged 18 years and over. The demographic weighting variables were obtained from the 2020 Current Population Survey. 

### 2.3. COVID-19 Physical and Mental Health Symptoms

Our primary outcomes for this analysis were physical and mental health symptoms experienced in the last seven days. To evaluate physical symptoms, we used participants’ responses (yes/no) to the following question: “Have you experienced any of the following symptoms in the past 7 days or not?” Of the 17 options, participants were able to select all that apply: fever, chills, runny or stuffy nose, chest congestion, skin rash, cough, sore throat, sneezing, muscle or body aches, headaches, fatigue or tiredness, shortness of breath, abdominal discomfort, nausea or vomiting, diarrhea, changed or loss sense of taste or smell, and loss of appetite. To evaluate mental health symptoms, we used participants’ responses (yes/no) to the following questions: “In the past 7 days, how often have you?: Felt nervous, anxious, or on edge; Felt depressed; Felt lonely; Felt hopeless about the future; Had physical reactions such as sweating trouble breathing, nausea or a pounding heart when thinking about your experience with the coronavirus pandemic.” Participants chose from the following list of options for each mental health symptom: Not at all or less than 1 day, 1–2 days, 3–4 days, 5–7 days.

### 2.4. Allergy History and Covariates

The primary predictor for this analysis was participants’ self-report of a physician diagnosis of an allergy. Participants were asked to reply “yes, no, or not sure” to the following question: “Has a doctor or other health care provider ever told you that you have any of the following: allergies.” The following covariates were included in the multivariable analyses: age categories (18–29, 30–44, 25–59, 60+), gender (male, female), race/ethnicity categories (non-Hispanic White, non-Hispanic Black, Hispanic, non-Hispanic Other), insurance status (yes/no), asthma diagnosis (yes/no), and obesity status (yes/no).

## 3. Data Analysis

Descriptive statistics are displayed in percentages among all respondents, unless otherwise labeled, and include a margin of error of +/−3·0 percentage points at the 95% confidence intervals among all adults. Chi-squared tests were used for univariate comparison of categorical variables including age, gender, race/ethnicity and obesity. Logistic regression was used to calculate the odds ratios and 95% confidence intervals associated with reporting “yes” (versus no) to COVID-19 related physical symptoms by allergy diagnosis. The multivariable logistic regression models included adjustments for age, gender, race/ethnicity, insurance status, asthma, and obesity. For mental health outcomes, we used multinomial logistic regression to compare mental health symptoms experienced in the last 7 days among adults with allergies to those without allergies after adjustment for age, gender, race/ethnicity, insurance status, history of asthma diagnosis, and obesity status. The Type I error was maintained at 5%. Based on the exploratory nature of this analysis, we did not include an adjustment for multiple comparisons [14,15]. Less than 1% of participants reported having received a physician diagnosis of COVID-19. We conducted a sensitivity analysis excluding participants with a diagnosis of COVID-19 and the results were unchanged. We present the results utilizing the full participant sample. 

All statistical analyses were conducted using Stata IC 15 (StataCorp LLC, College Station, TX, USA). Sampling weights were applied to provide results that were nationally representative of the U.S. adult population. Sampling weights were generated by analysts at NORC and included in the public use dataset. As recommended in the NORC methodology report, we utilized the combined sampling weight for any statistical analyses which include data Waves 1, 2, and 3. More detailed information can be found in the COVID-19 Impact Methodology Report. 

## 4. Results

Demographic characteristics for the 10,760 participants are included in Table 1, and distribution of demographic characteristics (e.g., age, race/ethnicity, sex) did not differ across allergy history. Within the overall sample, 43% (*n* = 4621) of participants reported a lifetime history of allergies. Participants without allergies had a higher proportion of uninsured than those with allergies (10.9, 95% CI 9.8, 12.2 compared to 5.8, 95% CI 5.0, 6.8, respectively).

As shown in Table 2, in the past 7 days, adults with allergies were significantly more likely to report physical symptoms compared to adults without allergies including fever (21.7% compared to 12.4%, χ^2^(1, 10359) = 158.38, *p* < 0.001), chills (18.4% compared to 10.8%, χ^2^(1, 10379) = 121.29, *p* < 0.001), runny or stuffy nose (91.3% compared to 10.3%, χ^2^(1, 10370) = 167.53, *p* < 0.001), chest congestion (20.3% compared to 10.6%, χ^2^(1, 10382) = 188.69, *p* < 0.001), skin rash (20.9% compared to 10.9%, χ^2^(1, 10380) = 193.69, *p* < 0.001), and loss or change in sense of taste or smell (17.9% compared to 9.6%, χ^2^(1, 10383) = 154.21, *p* < 0.001). 

In the multivariable model (Table 3), after adjusting for age, sex, race, insurance status, asthma, and obesity, adults with allergies were significantly more likely to report all physical symptoms compared to adults without allergies including fever (aOR 1.7, 95% CI 1.44–1.99), cough (aOR 1.9, 95% CI 1.60–2.26), shortness of breath (aOR 2.04, 95% CI 1.71–2.43), and loss of taste or sense of smell (aOR 1.9, 95% CI 1.58–2.28). 

Compared to adults without allergies, adults with allergies more frequently reported feeling anxious or nervous for 5–7 days out of the last 7 days (8.3% compared to 5.2%, χ^2^(3, 10456) = 133.01, *p* < 0.001), feeling depressed (8.4% compared to 6.2%, χ^2^(3, 10449) = 106.52, *p* < 0.001), feeling lonely (8.8% compared to 5.6%, χ^2^(3, 10439) = 94.13, *p* < 0.001), feeling hopeless (8.8% compared to 6.2%, χ^2^(3, 10440) = 91.73, *p* < 0.001), and having a physical reaction due to COVID-19 related stress (2.1% compared to 0.6%, χ^2^(3, 10463) = 138.49, *p* < 0.001) (Table 4).

In multivariable models (Table 5), compared to adults without allergies, adults with allergies were significantly more likely to report feeling nervous (cOR 1.37, 95% CI 1.18–1.58), depressed (cOR 1.33, 95% CI 1.16–1.54), lonely (cOR 1.22, 95% CI 1.06, 1.41), and having a physical reaction (cOR 1.57, 1.23–1.99) 1–2 days per week. In multivariable models, adults with allergies were significantly more likely to report feeling nervous (cOR 1.34, 95% CI 1.13, 1.60), depressed (cOR 1.32, 95% CI 1.11–1.57), lonely (cOR 1.23, 95% CI 1.04–1.47), hopeless (cOR 1.44, 95% CI 1.21–1.72), or having a physical reaction (cOR 2.01, 95% CI 1.44–2.82) on 3 or more days per week, compared to those without allergies. 

## 5. Discussion

We observed that during the COVID-19 pandemic, adults with allergies are more likely to report physical and mental health symptoms compared to individuals without allergies. The mental health findings are consistent with prior published studies highlighting increased reports of anxiety, depression, and other mental health symptoms among adults with pre-existing chronic health conditions, including allergies. As the study is a secondary data analysis of the COVID-19 Impact survey, we acknowledge that the study is likely to be overpowered to examine these associations; thus, effect sizes of observed associations in addition to statistical significance should be considered. 

In our study, we observed that adults with self-reported allergies were more likely to report several physical symptoms that have been associated with COVID-19 disease, including fever, loss of sense taste or smell, and cough. Prior studies have evaluated the associations of allergic conditions, including allergic rhinitis, with SARS-CoV-2 infection and risk of adverse clinical outcomes of COVID-19. Early reports of COVID-19 patients from Wuhan, China suggested allergic conditions were not risk factors of SARS-CoV-2 infection, rather older age, and high number of comorbid conditions [16]. However, more recently, in South Korea, data from a nationwide insurance claims database showed that allergic rhinitis was associated with an increased likelihood of SARS-CoV-2 test positivity and worse clinical outcomes of COVID-19 [17]. There are several pathophysiological mechanisms that suggest allergies could increase risk of SARS-CoV-2 infection [18]. The respiratory virus provokes local inflammatory cascade response inducing the productive of cytokines that can worsen the symptoms associated with asthma and allergic conditions [19]. Additionally, patients with allergies have impaired secretion of innate interferons which can increase their risk of developing respiratory viral infections [18]. Our data are limited as details regarding type of allergy or allergic condition are not available, although reliability of self-reported allergic conditions has been previously established in other cohorts [20]. We were able to adjust for self-reported asthma in our models, evaluating physical and mental health symptoms to adjust for the potential confounding effects of a comorbid asthmatic condition. 

Our results also show that adults with allergies are experiencing mental health symptoms during the pandemic, similar to other adults with chronic conditions [21]. As the clinical manifestations of allergic conditions, specifically allergic rhinitis, are similar to the common symptoms of COVID-19, adults with allergies may be experiencing anxiety related to their symptoms. Our prior work shows that when compared to those without allergies, adults with allergies were more likely to engage in several COVID-19 preventive behaviors including canceling or postponing activities, wearing a face mask, avoiding public or crowded places, maintaining six feet of social distance, washing or sanitizing their hands, and avoiding contact with high-risk people [22]. Increased adherence to COVID-19 preventive behaviors further demonstrates the potential mental health impact of the pandemic on this group as social isolation may lead to depression and hopelessness [23], as we observed in our study. 

These findings have important implications given the overlapping clinical manifestations between allergic conditions and COVID-19. Additionally, allergies as well as COVID-19 have been independently linked to mental health symptoms [24,25,26]. Further complicating this picture is that the COVID-19 pandemic co-existed with the Spring allergy season, and therefore presented diagnostic and treatment challenges for allergy physicians. Longitudinal studies are clearly necessary to determine how to best discern between COVID-19 and allergic conditions, while also considering patients that might have both.

## Figures and Tables

**Table 1:** Characteristics of COVID Impact Survey respondents (*n* = 10,760), a nationally representative survey of the US, stratified by allergy diagnosis (April–June 2020).

	Total		Adults with Allergies		Adults without Allergies	
	Col %	95% CI	Col %	95% CI	Col %	95% CI
**Age**						
18–29	20.1	18.9, 21.4	19.9	18.0, 21.9	20.3	18.7, 22.0
30–44	25.5	24.5, 26.6	25.2	23.7, 26.8	25.8	24.3, 27.3
45–59	24.4	23.3, 25.6	26.1	24.4, 27.8	23.2	21.7, 24.6
60+	29.9	28.8, 31.1	28.8	27.2, 30.6	30.8	29.2, 32.4
**Sex**						
Male	47.9	46.6, 49.3	41.9	39.9, 43.9	52.6	50.9, 54.4
Female	52.1	50.7, 53.4	58.1	56.1, 60.1	47.4	45.6, 49.1
Race/Ethnicity **						
White, NH	61.7	60.4, 63.0	64.7	62.7, 66.7	59.3	57.5, 61.1
Black, NH	11.9	11.1, 12.8	10.4	9.2, 11.6	13.1	11.9, 14.3
Hispanic	16.4	15.3, 17.5	15.6	14.0, 17.3	17	15.6, 18.5
Asian, NH	5	4.3, 5.8	4.2	3.3, 5.4	5.6	4.6, 6.7
Other, NH	3.5	3.1, 4.0	3.8	3.2, 4.5	3.3	2.8, 3.9
**Asthma comorbidity**						
Yes	14.7	13.8, 15.7	11.4	10.6, 12.3	3.3	2.8, 3.9
No	85.3	84.3, 86.2	32.1	30.8, 33.3	53.2	51.2, 54.5
**Overweight or Obesity comorbidity**						
Yes	33.8	32.5, 35.0	17.7	16.7, 18.7	16.1	15.1, 17.1
No	66.2	65.0, 67.5	26	24.8, 27.2	40.2	38.9, 41.6
**Insurance Type or Health Coverage Plans**						
Insured	91.3	90.5, 92.1	94.2	93.2, 95.0	89.1	87.8, 90.2
No insurance	8.7	7.9, 9.5	5.8	5.0, 6.8	10.9	9.8, 12.2

** Missing values for race/ethnicity were excluded from the table due to small sample size.

**Table 2:** Report of COVID-19 physical symptoms in the past 7 days among COVID Impact Survey respondents (*n* = 10, 760), stratified by allergy diagnosis (April–June 2020).

	Total		Adults without Allergies		Adults with Allergies		*p*-Value *
	Col %	95% CI	Col %	95% CI	Col %	95% CI	
**Report of fever in the past 7 days**							<0.001
Yes (*n* = 1623)	16.5	15.5, 17.5	12.4	11.3, 13.7	21.7	20.0, 23.4	
No (*n* = 8736)	83.5	82.5, 84.5	87.6	86.3, 88.7	78.3	76.6, 80.0	
**Report of chills in the past 7 days**							<0.001
Yes (*n* = 1474)	14.1	13.2, 15.1	10.8	9.7, 12.1	18.4	16.9, 20.1	
No (*n* = 8905)	85.9	84.9, 86.8	89.2	87.9, 90.3	81.6	79.9, 83.1	
**Report of runny or stuffy nose in the past 7 days**							<0.001
Yes (*n* = 1453)	14.2	13.3, 15.2	10.3	9.2, 11.5	19.3	17.7, 20.9	
No (*n* = 8917)	85.8	84.8, 86.7	89.7	88.5, 90.8	80.7	79.1, 82.3	
**Report of chest congestion in the past 7 days**							<0.001
Yes (*n* = 1546)	14.8	13.9, 15.8	10.6	9.5, 11.9	20.3	18.8, 21.9	
No (*n* = 8836)	85.2	84.2, 86.1	89.4	88.1, 90.5	79.7	78.1, 81.2	
**Report of skin rash the past 7 days**							<0.001
Yes (*n* = 1525)	15.3	14.3, 16.3	10.9	9.8, 12.2	20.9	19.3, 22.5	
No (*n* = 8855)	84.7	83.7, 85.7	89.1	87.8, 90.2	79.1	77.5, 80.7	
**Report of cough in the past 7 days**							<0.001
Yes (*n* = 1454)	13.5	12.6, 14.4	9.4	8.4, 10.5	18.7	17.2, 20.3	
No (*n* = 8934)	86.5	85.6, 87.4	90.6	89.5, 91.6	81.3	79.7, 82.8	
**Report of sore throat in the past 7 days**							<0.001
Yes (*n* = 1434)	13.6	12.7, 14.5	9.2	8.3, 10.3	19.2	17.7, 20.8	
No (*n* = 8965)	86.4	85.5, 87.3	90.8	89.7, 91.7	80.8	79.2, 82.3	
**Report of sneezing in the past 7 days**							<0.001
Yes (*n* = 1396)	13.4	12.5, 14.3	9.7	8.7, 10.9	18.1	16.7, 19.7	
No (*n* = 8962)	86.6	85.7, 87.5	90.3	89.1, 91.3	81.9	80.3, 83.3	
**Report of muscle or body aches in the past 7 days**							<0.001
Yes (*n* = 1373)	13.1	12.2, 14.0	8.8	7.9, 9.9	18.6	17.1, 20.1	
No (*n* = 9019)	86.9	86.0, 87.8	91.2	90.1, 92.1	81.4	79.9, 82.9	
**Report of headaches in the past 7 days**							<0.001
Yes (*n* = 1502)	14.2	13.3, 15.2	10.4	9.4, 11.5	19.2	17.6, 20.8	
No (*n* = 8911)	85.8	84.8, 86.7	89.6	88.5, 90.6	80.8	79.2, 82.4	
**Report of fatigue or tiredness in the past 7 days**							<0.001
Yes (*n* = 1354)	13.7	12.7, 14.6	10.3	9.2, 11.5	18	16.5, 19.6	
No (*n* = 9040)	86.3	85.4, 87.3	89.7	88.5, 90.8	82	80.4, 83.5	
**Report of shortness of breath in the past 7 days**							<0.001
Yes (*n* = 1342)	12.4	11.6, 13.3	8.3	7.4, 9.3	17.7	16.2, 19.2	
No (*n* = 9044)	87.6	86.7, 88.4	91.7	90.7, 92.6	82.3	80.8, 83.8	
**Report of abdominal discomfort in the past 7 days**							<0.001
Yes (*n* = 1224)	11	10.2, 11.8	7.8	7.0, 8.8	15	13.7, 16.4	
No (*n* = 9181)	89	88.2, 89.8	92.2	91.2, 93.0	85	83.6, 86.3	
**Report of nausea or vomiting in the past 7 days**							<0.001
Yes (*n* = 1342)	12.6	11.7, 13.5	9.3	8.3, 10.3	16.8	15.4, 18.3	
No (*n* = 9056)	87.4	86.5, 88.3	90.7	89.7, 91.7	83.2	81.7, 84.6	
**Report of diarrhea in the past 7 days**							<0.001
Yes (*n* = 1334)	12.8	11.9, 13.7	8.9	7.9, 9.9	17.8	16.3, 19.4	
No (*n* = 9055)	87.2	86.3, 88.1	91.1	90.1, 92.1	82.2	80.6, 83.7	
**Report of change or loss of taste or smell in the past 7 days**							<0.001
Yes (*n* = 1359)	13.2	12.3, 14.2	9.6	8.5, 10.8	17.9	16.4, 19.5	
No (*n* = 9024)	86.8	85.8, 87.7	90.4	89.2, 91.5	82.1	80.5, 83.6	
**Report of loss of appetite in the past 7 days**							<0.001
Yes (*n* = 1257)	11.6	10.8, 12.5	7.9	7.1, 8.9	16.4	15.0, 17.9	
No (*n* = 9136)	88.4	87.5, 89.2	92.1	91.1, 92.9	83.6	82.1, 85.0	

* *p*-value is based on a chi-squared test to compare symptoms reported by self-reported allergy status.

**Table 3:** Odds and 95% CI of adults with allergies reporting a physical symptom within the last 7 days.

Symptom	Adjusted Odds Ratio	95% Confidence Intervals
Fever	1.70	1.44–1.99
Chills	1.74	1.46–2.08
Runny or stuffy nose	1.74	1.46–2.06
Chest congestion	1.75	1.48–2.06
Skin rash	1.89	1.61–2.23
Cough	1.90	1.60–2.26
Sore throat	1.90	1.60–2.26
Sneezing	1.66	1.39–1.98
Muscle or body aches	1.97	1.66–2.34
Headaches	1.85	1.56–2.18
Fatigue	1.65	1.40–1.94
Shortness of breath	2.04	1.71–2.43
Abdominal discomfort	1.84	1.55–2.19
Nausea	1.65	1.38–1.98
Diarrhea	1.98	1.66–2.36
Lost taste or sense of smell	1.90	1.58–2.28
Appetite	1.86	1.56–2.22

Models adjusted for: Age, gender, race, insurance status, asthma, and obesity.

**Table 4:** Report of COVID-19 mental health symptoms in the past 7 days among COVID Impact Survey respondents (*n* = 10, 760), stratified by allergy diagnosis (April–June 2020).

	Total		Adults without Allergies		Adults with Allergies		*p*-Value *
	Col %	95% CI	Col %	95% CI	Col %	95% CI	
**Felt nervous, anxious, or on edge**							<0.001
Not at all or less than 1 day (*n* = 6417)	62.1	60.8, 63.4	66.7	65.0, 68.4	56.1	54.1, 58.1	
1–2 days (*n* = 2359)	21.7	20.7, 22.8	19.2	17.9, 20.5	25	23.3, 26.8	
3–4 days (*n* = 984)	9.6	8.8, 10.5	8.9	7.9, 10.1	10.5	9.3, 11.8	
5–7 days (*n* = 696)	6.5	5.9, 7.2	5.2	4.5, 6.0	8.3	7.3, 9.5	
**Felt depressed**							<0.001
Not at all or less than 1 day (*n* = 6375)	61.2	59.9, 62.5	65.5	63.8, 67.2	55.7	53.7, 57.7	
1–2 days (*n* = 2367)	21.9	20.8, 23.0	20	18.6, 21.3	24.3	22.6, 26.1	
3–4 days (*n* = 1011)	9.8	9.0, 10.6	8.4	7.4, 9.5	11.5	10.3, 12.9	
5–7 days (*n* = 696)	7.1	6.5, 7.9	6.2	5.4, 7.1	8.4	7.3, 9.6	
**Felt lonely**							<0.001
Not at all or less than 1 day (*n* = 6440)	61.2	59.9, 62.5	65.1	63.3, 66.8	56.3	54.4, 58.3	
1–2 days (*n* = 2324)	22.4	21.3, 23.5	20.6	19.2, 22.2	24.6	22.9, 26.3	
3–4 days (*n* = 987)	9.4	8.7, 10.2	8.7	7.7, 9.9	10.3	9.3, 11.5	
5–7 days (*n* = 688)	7	6.3, 7.7	5.6	4.8, 6.5	8.8	7.6, 10.1	
**Felt hopeless about the future**							<0.001
Not at all or less than 1 day (*n* = 6334)	61.3	60.0, 62.6	64.8	63.0, 66.4	56.8	54.8, 58.7	
1–2 days (*n* = 2499)	23.4	22.3, 24.5	22.5	21.1, 24.1	24.5	22.9, 26.2	
3–4 days (*n* = 873)	8	7.3, 8.8	6.5	5.7, 7.5	9.9	8.7, 11.2	
5–7 days (*n* = 734)	7.3	6.7, 8.1	6.2	5.4, 7.2	8.8	7.8, 10.0	
**Had physical reaction**							<0.001
Not at all or less than 1 day (*n* = 9436)	90.5	89.7, 91.2	93.3	92.5, 94.1	86.8	85.3, 88.1	
1–2 days (*n* = 645)	5.8	5.2, 6.4	4.3	3.7, 5.1	7.6	6.7, 8.7	
3–4 days (*n* = 255)	2.5	2.1, 2.9	1.7	1.3, 2.1	3.5	2.7, 4.4	
5–7 days (*n* = 127)	1.3	1.0, 1.6	0.6	0.5, 0.9	2.1	1.6, 2.9	

* *p*-value is based on a chi-squared test to compare symptoms reported by self-reported allergy status.

**Table 5:** Conditional odds ratios and 95% CI of mental health symptoms of adults with allergies.

	Never (Reference)	1–2 Days/Week		3+ Days/Week	
		Conditional OR	95% CI	Conditional OR	95% CI
Felt nervous/anxious/on edge	1.00	1.37	1.18–1.58	1.34	1.13–1.60
Felt depressed	1.00	1.33	1.16–1.54	1.32	1.11–1.57
Felt lonely	1.00	1.22	1.06–1.41	1.23	1.04–1.47
Felt hopeless	1.00	1.13	0.98–1.29	1.44	1.21–1.72
Had physical reaction	1.00	1.57	1.23–1.99	2.01	1.44–2.82

Models adjusted for: Age, gender, race, insurance status, asthma, and obesity.

## Data Availability

Publicly available datasets were analyzed in this study. The data can be found here: https://www.covid-impact.org/results.

## Abbreviations

COVID-19	coronavirus disease 2019
SARS-CoV-2	severe acute respiratory syndrome coronavirus 2
U.S.	United States
cOR	conditional odds ratio
aOR	adjusted odds ratios
95% CI	95% confidence intervals
